# Real world clinical practice, efficacy and safety of esketamine nasal spray in MDD patients: the French ELLIPSE study

**DOI:** 10.1192/j.eurpsy.2025.682

**Published:** 2025-08-26

**Authors:** P.-M. Llorca, L. Mékaoui, M. Rotharmel, P. de Maricourt, C. Wicart, E. Gaudre-Wattinne, J. Dupin

**Affiliations:** 1 Department of Psychiatry, CHU Clermont-Ferrand, University of Clermont Auvergne, CNRS, Clermont Auvergne INP, Institute Pascal (UMR 6602), Clermont-Ferrand; 2 Mental and Brain Illness Clinic, Sainte Anne Hospital GHU Paris – Psychiatry and Neurosciences, Paris; 3 University Department of Psychiatry, Therapeutic Centre of Excellence, Institute of Psychiatry – Rouvray Hospital Centre, Sotteville-lès-Rouen; 4 Sainte Anne Hospital GHU Paris – Psychiatry and Neurosciences, Paris; 5 Medical Affairs, Janssen Cilag, Issy-les-Moulineaux, France

## Abstract

**Introduction:**

Major depressive disorder (MDD) is the leading cause of disability worldwide. About one third of patients with MDD fails to achieve remission despite treatment and can be considered as Treatment-Resistant patients. Esketamine nasal spray (ESK) is indicated since 2019 in US and Europe for adults with Treatment-Resistant Depression (TRD), who have not responded to at least two different treatments with antidepressants in the current moderate to severe depressive episode. ELLIPSE is the first prospective observational study on ESK in real world conditions in France.

**Objectives:**

The aim of the study is to describe the profile of all patients treated with ESK in real-world clinical practice, the modalities of the use of ESK, the patient management at the site of care and the efficacy and safety outcomes during 12 months after ESK treatment initiation for MDD patients.

**Methods:**

ELLIPSE is a French prospective, multicenter, non-interventional study designed to describe patients presenting MDD treated with ESK, and for whom the decision to prescribe ESK is independent of the study. Data were collected from physicians as part of routine clinical practice and from patients via self-questionnaires.

**Results:**

Thirty-one sites have included 211 MDD patients treated with ESK. The analysis will describe patient profiles (sociodemographic and disease characteristics, medical history, comorbidities), use of ESK at initiation and during study (reason for initiation, posology, frequency, administration, surveillance, treatment duration), safety profile (percentage of reported adverse events) and efficacy (MADRS response and remission rates over 12 months). The study results will be available in January 2025 and will be detailed in the poster presented at the conference.

**Conclusions:**

ELLIPSE is the first large prospective French study providing real world evidence on patients treated with ESK, including patient’s profile, condition of use of ESK treatment and follow-up efficacy and safety data. This study should confirm that ESK has its place in therapy for the treatment of MDD.

**Disclosure of Interest:**

P.-M. Llorca Consultant of: Abbvie, Boehringer-Ingelheim, Eisai, Ethypharm, HAC, Janssen, Karla Therapeutics, Lilly, Lundbeck, MSD, Neuraxpharm, Newron, Novartis, Otsuka, Roche, Sanofi, Teva. He provided expert testimony for Janssen, Otsuka., L. Mékaoui Consultant of: Janssen, M. Rotharmel Consultant of: Janssen, P. de Maricourt Consultant of: Janssen, C. Wicart Employee of: Janssen, E. Gaudre-Wattinne Employee of: Janssen, J. Dupin Employee of: Janssen.

